# Supplementary material for: Multi-Trait Exome-Wide Association Study of Back Pain-Related Phenotypes
Source: Genes (Basel). 2023 Oct 19;14(10):1962. doi: 10.3390/genes14101962 (PMC10606006; doi:10.3390/genes14101962)
Supplement: Supplementary file 1 [file genes-14-01962-s001.zip › genes-2650519-supplementary.pdf]

SUPPLEMENTARY INFORMATION

Multi-Trait Exome-Wide Association Study of Back Pain-Related Phenotypes

Irina V. Zorkoltseva , Elizaveta E. Elgaeva , Nadezhda M. Belonogova ,
Anatoliy V. Kirichenko , Gulnara R. Svishcheva , Maxim B. Freidin , Frances M. K. Williams ,
Pradeep Suri , Yakov A. Tsepilov and Tatiana I. Axenovich

SUPPLEMENTARY METHODS

SHAHER analysis

SHAHER analysis includes two steps. On the first step, the alpha coefficients of the linear combination of the original traits are calculated by the maxSH method. The input data for this method are phenotypic and genetic correlations between the original traits and their SNP-based heritabilities.

Pairwise phenotypic correlations between the three original back pain (BP) traits were assessed in a subsample of non-relatives only (307,876 white individuals with chronic back pain (CBP), dorsalgia, and intervertebral disc disordeds (IDD) status information. Genetic correlations between the original traits and SNP-based heritabilities were calculated using LD score regression (Bulik-Sullivan et al., 2015) on GWAS results. GWAS summary statistics for the original traits were calculated on imputed genotypes using white British individuals (N = 449,136) by fastGWA-GLMM as described in EWAS analysis and were filtered to keep SNVs with imputation quality INFO > 0.8 and MAF > 5×10^{-6}. Total number of SNPs was 19,405,718 after filtration. Prior to the genetic correlations and heritability estimation, the summary statistics were reformatted using the munge() function with the default settings from GenomicSEM v0.0.2 R package (Grotzinger et al., 2019).

On the second step of SHAHER, the summary statistics of multi-trait SGIT (shared genetic impact trait) are calculated using the sumCOT method. The input data for sumCOT are the alpha

coefficients and the summary statistics obtained by analysis of associations between the original traits and exome sequenced genotypes.

MAC cutoff for collapsing ultra-rare variants

We checked that the minor allele count (MAC) ≥ 10 cutoff proposed by Zhou et al. (2022) is optimal for the data analyzed. Initially, we estimated the inflation factors for all traits and all variants with MAF < 0.01. These estimates differed from the expected values of 1 for all traits (Table 1). Then we calculated the inflation factors for variants with different MAC cutoffs. Table summarizes the inflation factors for all traits after different cutoffs. As can be seen, the MAC cutoff decreased the inflation factor if it initially was greater than 1, and increased it if it was less than 1. The optimal MAC cutoff was different for different traits. We must select a single threshold for all traits analyzed because we used them for multi-trait building. We selected the MAC > 10 cutoff, which is a mean value for all traits.

Table. Inflation factors for different MAC cutoffs.

Phenotype	All variants	MAC[a] > 5	MAC > 10	MAC > 15	MAC > 20
CBP[b]	1.130	1.171	1.058	1.024	1.027
IDD[c]	0.172	0.423	0.756	0.981	1.117
Dorsalgia	0.333	0.750	1.116	1.251	1.396

[a] Minor allele count
[b] Chronic back pain
[c] Intervertebral disc disorder

References

Bulik-Sullivan BK, Loh PR, Finucane HK, et al. LD Score regression distinguishes confounding from polygenicity in genome-wide association studies. *Nat Genet*. 2015;47(3):291-295.

Grotzinger AD, Rhemtulla M, de Vlaming R, et al. Genomic structural equation modelling provides insights into the multivariate genetic architecture of complex traits. *Nat Hum Behav*. 2019;3(5):513-525.

Zhou W, Bi W, Zhao Z, et al. SAIGE-GENE+ improves the efficiency and accuracy of set-based rare variant association tests. *Nat Genet*. 2022;54(10):1466-1469.

Supplementary Tables

Table S1. Characteristics of the patients with different BP-related phenotypes

Phenotype	Cases	Age mean (min-max)	Gender (Male/ Female)
CBP	32040	57.52 (40.0 - 72.9)	14214/17826
Dorsalgia	6233	58.49 (40.3 – 78.3)	2578/3655
IDD	3183	57.87 (40.2 – 77.0)	1334/1849

Table S2. Numbers of collapsed ultra-rare variants (MAC ≤ 10) and other non-ultra-rare variants (MAC > 10) in different variant annotations

Annotation	MAC ≤ 10	MAC > 10
LoF	312,649	33,420
LoF + missense	3,357,904	548,059
LoF + protein coding	4,667,652	829,747
All intragenic	9,558,446	1,551,580

SUPPLEMENTARY FIGURES

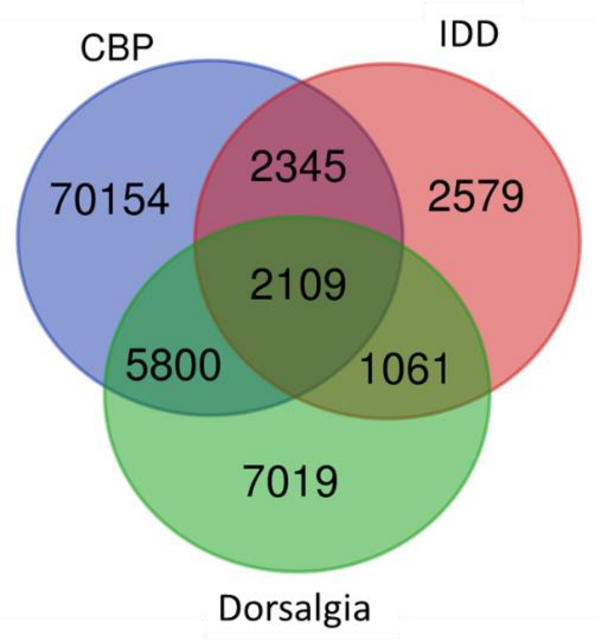

Figure S1. Venn diagram showing the distribution of phenotypes among individuals in 500K data set

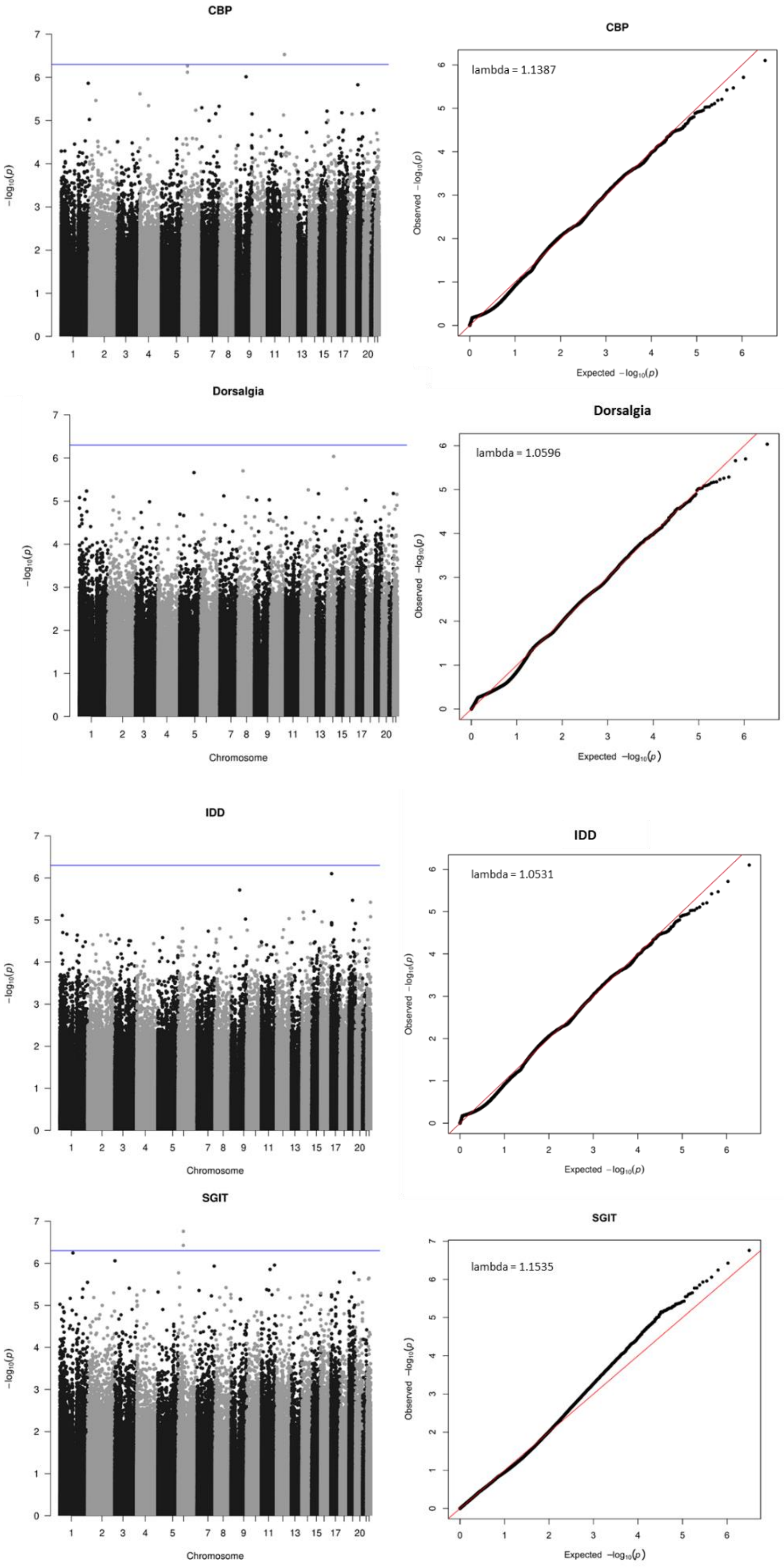

Figure S2. Manhattan (left) and QQ (right) plots for single-point association analyses of different traits using exome sequencing data. The plots represent the −log10 transformed p-values. The horizontal blue line represents the genome-wide suggestive significance (p-value = 5×10^{-7}).

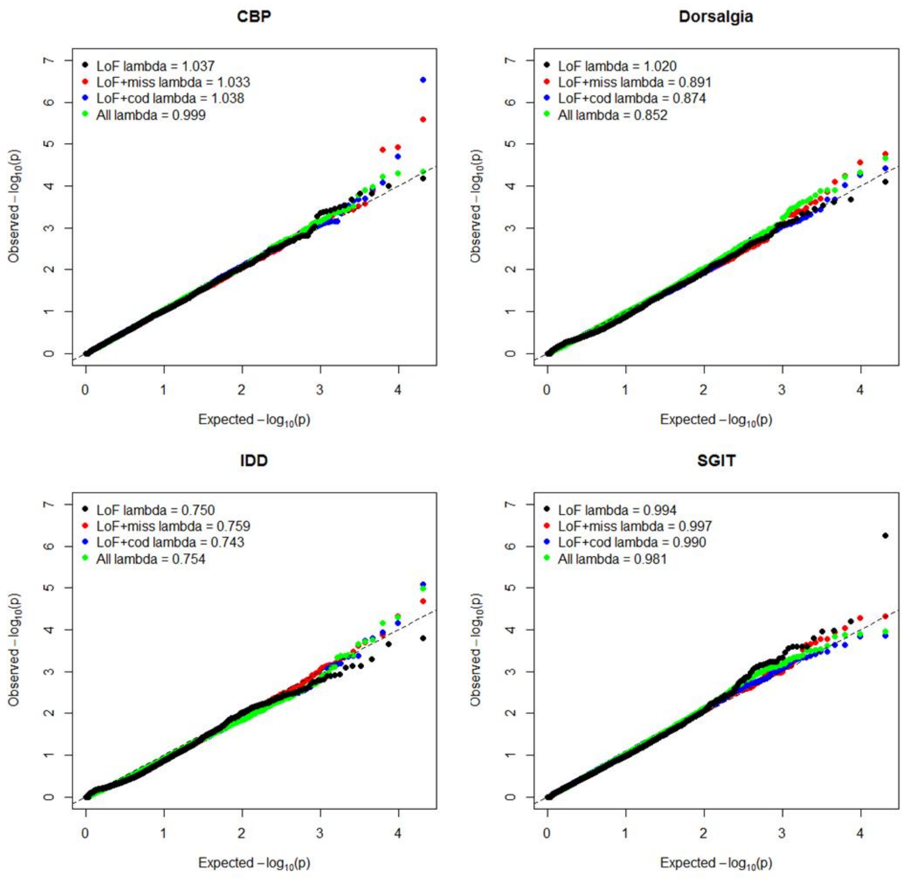

Figure S3. QQ-plots for gene-based analyses of different traits and variant annotations using exome sequencing data
